# Effects of Long-Term Exposure to Zinc Oxide Nanoparticles on Development, Zinc Metabolism and Biodistribution of Minerals (Zn, Fe, Cu, Mn) in Mice

**DOI:** 10.1371/journal.pone.0164434

**Published:** 2016-10-12

**Authors:** Chao Wang, Jianjun Lu, Le Zhou, Jun Li, Jiaman Xu, Weijian Li, Lili Zhang, Xiang Zhong, Tian Wang

**Affiliations:** 1 College of Animal Science and Technology, Nanjing Agricultural University, Nanjing, People’s Republic of China; 2 Institute of Feed Science, College of Animal Science, Zhejiang University, Key Laboratory of Molecular Animal Nutrition, Ministry of Education, Hangzhou Zhejiang Province, People’s Republic of China; Institute of Materials Science, GERMANY

## Abstract

Zinc oxide nanoparticles (nano-ZnOs) are widely used and possess great potentials in agriculture and biomedicine. It is inevitable for human exposure to these nanoparticles. However, no study had been conducted to investigate the long term effects of nano-ZnOs. This study aimed at investigating effects of nano-ZnOs on development, zinc metabolism and biodistribution of minerals (Zn, Fe, Cu, and Mn) in mice from week 3 to 35. After the characteristics of nano-ZnOs were determined, they were added into the basal diet at 0, 50, 500 and 5000 mg/kg. Results indicated that added 50 and 500 mg/kg nano-ZnOs showed minimal toxicity. However, 5000 mg/kg nano-ZnOs significantly decreased body weight (from week 4 to 16) and increased the relative weights of the pancreas, brain and lung. Added 5000 mg/kg nano-ZnOs significantly increased the serum glutamic-pyruvic transaminase activity and zinc content, and significantly enhanced mRNA expression of zinc metabolism-related genes, including metallothionein 1(32.66 folds), metallothionein 2 (31.42 folds), ZIP8 (2.21folds), ZIP14 (2.45 folds), ZnT1 (4.76 folds), ZnT2 (6.19 folds) and ZnT4 (1.82 folds). The biodistribution determination showed that there was a significant accumulation of zinc in the liver, pancreas, kidney, and bones (tibia and fibula) after receiving 5000 mg/kg nano-ZnO diet, while no significant effects on Cu, Fe, and Mn levels, except for liver Fe content and pancreas Mn level. Our results demonstrated that long term exposure to 50 and 500 mg/kg nano-ZnO diets showed minimal toxicity. However, high dose of nano-ZnOs (5000 mg/kg) caused toxicity on development, and altered the zinc metabolism and biodistribution in mice.

## Introduction

In the last decades, nanotechnology has been developing fast and it is inevitable for human to expose to nanostructured materials, so more concerns have been spurred over the possible adverse effects of exposure to nanoparticles [1–3]. The key reason for the emerging development of nanotechnology could be attributed to the decreased particle size and increased surface area to volume ratio at the nanoscale which brings quantum mechanics and results in novel and enhanced potentials[4]. Owing to their high surface area, enhanced reactivity, and high chemical stability, metal oxide nanoparticles possess excellent potentials and are widely applied [5,6]. Among various metal oxides, zinc oxide nanoparticles (nano-ZnOs) are the best studied and most widely used nanostructured materials for their multifunctional physical and chemical properties and easy synthesis[5]. Nano-ZnOs can be prepared by various methods, such as the traditional high temperature solid state method, chemical precipitation, sol-gel synthesis, and hydrothermal method[7]. Currently, nano-ZnOs are widely used in personal care products (cosmetics and sunscreens), plastics, ceramics, glass, rubber, batteries, fire retardant [8]. It was also reported that nano-ZnOs showed greater effects on the inhibition of cancer cells than the existing technologies [5,6]. Owing to the high antibacterial activity, chemical stability and solubility, nano-ZnO shows great interests in the application in fields of food additives, packing and agriculture, and biomedicine [9–11]. Many factors could affect the antibacterial property of nano-ZnOs, including the pH precursor solution and reagents controlling the growth of nano-ZnOs, the morphology, particle size and so on [11,12].

As for the popular application of nano-ZnOs, the human is commonly exposed to such nanoparticles. It is an emerging need to investigate the toxicity of nano-ZnOs, which can easily enter cells and introduce oxidative stress, although it has been reported that nano-ZnOs exhibited high biocompatibility with human cells and high selectivity[11,13]. Some studies have been conducted to investigate the short term effects of nano-ZnOs in vitro and vivo. After an exposure of 0.5h to 72h, nano-ZnOs could cause acute cytotoxicity on various cell types, such as human epidermal cells [14], liver and retinal cells [15,16] and white blood cells [17] via interfering with zinc ion homeostasis or inducing oxidative stress. In vivo, inhalation exposure to nano-ZnOs for 3 days caused severe damage in liver and lung tissues [18]. Acute exposure to high dose ranges of nano-ZnOs (1–5 g/kg body weight) via oral treatment damaged the liver, spleen and pancreas in mice [19]. With the repeated dermal exposure for 28 days in Sprague-Dawley rats, nano-ZnOs showed dose-dependent toxicity on collagen in skin, potential reasons for which may be that the nano-ZnOs passed through skin due to their small size [20]. Oral treatment with nano-ZnOs for 14 days could disturb energy metabolism and cause mitochondria and cell membrane impairment in rat kidney, which may contribute to nano-ZnO induced nephrotoxicity [21]. Recently, it was reported that the oral doses for the study with 15-days repeated of nano-ZnOs were maternotoxic in the 200 mg/kg/day group, and embryotoxic in the 400 mg/kg/day group [22,23]. Despite of the significant acute effects of nano-ZnOs on mice and rats, the long term effects of nano-ZnOs on the development has not been reported. Moreover, it was supposed that the toxicity of nano-ZnOs could be due to interfering with the zinc ion hemostasis via enhanced absorption and transported to the target organs [17,20,24]. However, effects of nano-ZnOs on the zinc metabolism in small intestine are still unclear.

Therefore, our present study was conducted to evaluate the long term effects of doses of nano-ZnOs on the development, zinc metabolism, and mineral biodistribution (Zn, Fe, Cu, Mn) in different tissues (kidney, muscle, pancreas, liver, brain, testis and bone). Results of this study could provide additional relevant information to the safety evaluation and application of nano-ZnOs via long term oral exposure in mice.

## Material and Methods

Experiments were approved and conducted under the supervision of the Institutional Animal Care and Use Committee of Nanjing Agriculture University, China.

### Characterization of the nano-ZnOs

The nano-ZnOs provided by Hangzhou King Techina Technology CO., Ltd (Zhejiang, China) were characterized in the term of primary sizes, morphological and crystalline properties by using the Malvern Autosier (Malvern Instruments, Malvern, UK), and transmission electron microscope (TEM, JEM-200CX, Japan). The nano-ZnOs for TEM were suspended in methanol and were placed onto a carbon coated copper grid. The high resolution transmission electron microscopy (HR-TEM) was operated at 200 kV.

### Animal and experimental design

The basal mice diet and 48 healthy CD-ICR male mice (3-week old), weight about 11.50 g, were provided by Experimental Animal Center, Yangzhou University (Jiangsu, Chian). Mice were randomly divided into four groups, 12 mice per group. They were fed diets as follow: (1) the control group: basal diet; (2) 50 mg/kg nano-ZnO group: basal diet+50 mg/kg nano-ZnOs; (3) 500 mg/kg nano-ZnO group: basal diet+500 mg/kg nano-ZnOs; (4) 5000 mg/kg nano-ZnO group: basal diet+5000 mg/kg nano-ZnOs. Mice were housed in polypropylene cages and maintained in an animal house at 20±5°C and 12h light/dark cycle. Mice were fed with deionized water and *ad libitum*. The food and water supply, house temperature, behavior patterns, and clinial signs of toxicity (lethargy, coma, tremors, nausea, vomiting, diarrhoea, etc) will be checked twice a day (8:00 and 18:00). Once clinial sign occurred, mice would have been raised seperately. When mice became unconscious, they would have been sacrificed humanely with carbon dioxide. The duration of this experiment was 32 weeks, the body weight of each mouse was weighed every week and no mice died.

### Sample collection

At the end of the feeding trial, mice (a 12h fast) were weighed, and humanely sacrificed by carbon dioxide asphyxiation. Blood samples were centrifuged at 3000×g for 15min at 4°C to obtain serum, which was stored at -20°C until for analysis. The liver, kidney, brain, spleen, heart, pancreas, and testis were weighed and calculated relative organ weight (organ weight:body weight). The selected tissues and thigh muscle (rectus femoris muscle and vastus medialis muscle), and bone (tibia and fibula) samples were stored at -20°C. The 4cm jejunal samples were collected at a 7cm distance from the pyloric sphincter, quickly-frozen by immersion in liquid nitrogen and stored at -80°C until for analysis of zinc metabolism.

### Analysis of serum biochemical parameters

The concentrations of Zn, and the activities of glutamic oxalacetic transaminase (GOT), glutamic-pyruvic transaminase (GPT) and alkaline phosphatase (ALP) in serum were determined by corresponding commercial kits purchased from Nanjing Jiancheng Bioengineering Institute (Nanjing, China). These serum parameters were determined with the recommended procedure of each kit.

### Determination of mineral biodistribution

The mineral concentrations (Zn, Fe, Cu, and Mn) of tissues, including liver, kidney, brain, pancreas, testis, thigh muscle (rectus femoris muscle and vastus medialis muscle) and bones (tibia and fibula), were determined as described by Demirbaş [25] with some modifications. Briefly, tissues (0.5–1.5g) were digested using an acid mixture (HNO_3_:HClO_4_ = 4:1, v:v). The digest was brought to a volume of 25 ml with demineralized water. Blanks and standard solutions were prepared. After that, mineral concentrations were determined via inductively coupled plasma mass spectrometry (ICP-MS, USA).

### Determination of relative mRNA expression of Zn metabolism in jejunum

Total RNA of jejunum was extracted with Trizol reagents according to the manufacturer’s introduction (Invitrogen, USA). After that the RNA quality was verified by nano-drop 2000 (ratios of absorption including 260/280 nm and 260/230 nm between 1.90 and 2.05) and by agarose gel electrophoresis, 2 μg RNA was incubated at 72°C with Random Primer (Promega, Belgium) for 5 min and incubated for 1 h with reverse transcription mixture (Takara, Dalian, China), including 5×M-MLV-RT buffer, M-MLV reverse transcriptase and dNTPs. Finally, the reverse transcription was inactivated at 90°C for 10 min.

In this study, Zn metabolism-related gene expression of metallothionein 1 (MT1), metallothionein 2 (MT2), solute carrier family 39 member 8 (ZIP8), solute carrier family 39 member 8 (ZIP14), solute carrier family 30 member 1 (ZnT1), solute carrier family 30 member 2 (ZnT2), and solute carrier family 30 member 4 (ZnT4) in jejunum were determined. Gene-specific primers were listed in Table 1 and were synthesized by Invitrogen Biotech Co. LTD. (Shanghai, China). β-actin was chosen as the housekeeping gene. Reverse transcription polymerase chain reaction (RT-PCR) assays were conducted by utilization of ABI 7300 RT-PCR system (Applied Biosystems, Foster City, CA) with a SYBR Premix Ex Taq^TM^ Kit (TakaRa Biotechnology Co. Ltd, Dalian, China) according to the manufacturer’s instructions. The relative mRNA expression was determined with ABI software and calculated with the 2^-ΔΔCt^ as described by Livak, Schmittgen [26].

**Table 1:** Primers sequences used in quantitative real time PCR assays.

Genes	Accession No.	Primers	Sequences(5’—3’)	bp
**β-actin**	NM_007393.3	Forward	CTGTCCCTGTATGCCTCTG	218
		Reverse	ATGTCACGCACGATTTCC	
**ZIP 8**	NM_026228	Forward	AGGGCTTGGTCCCTGAGTTA	145
		Reverse	CCAGAGCATGGCAAGACTGA	
**ZIP 14**	NM_144808.4	Forward	ACGCCTCCCTTTTCCTTCTG	157
		Reverse	ATGCCAAAATGGACCGACCT	
**ZnT 1**	NM_009579.3	Forward	CAATTCCAACGGGCTGAAGG	213
		Reverse	ACCAAGGCATTCACGACCAC	
**ZnT 2**	NM_001039677	Forward	TGCCCAGGAGGAATTTGCTT	118
		Reverse	TGGCCTTGGTAAGCCATGTT	
**ZNT 4**	NM_001290993	Forward	CACCACCATTCTCACGCTCA	129
		Reverse	CAAGTCTCCCAAGGCGTGTA	
**MT1**	NM_013602	Forward	ATCTCGGAATGGACCCCAAC	297
		Reverse	ACTCGGTAGAAAACGGGGGT	
**MT 2**	NM_008630	Forward	GCATCTGCAAAGAGGCTTCC	127
		Reverse	GGAGAACGAGTCAGGGTTGT	

### Statistical analysis

The data were analyzed by Turkey’s multiple range test of SPSS statistical package for Windows (Version 20.0, SPSS Inc., Chicago, IL) and present as mean± SE. Effects of increasing level of nano-ZnOs were partitioned into linear, quadratic using polynomial trend analysis. *P* value below 0.05 was considered as statistical significant level.

## Results

### Characteristics of nano-ZnOs

**Fig 1:**
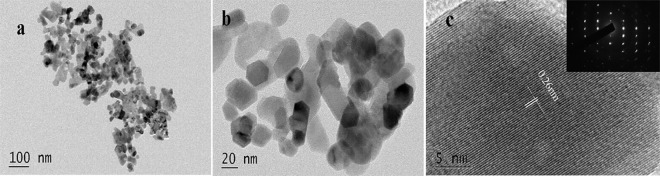
TEM image of nano-ZnOs: (a) low magnification image, (b) high magnification images, and (c) HR-TEM image.

In the present study, the average particle size of nano-ZnOs was 40.90±3.18 (n = 3). To confirm the size and determine the morphology and crystalline quality, nano-ZnOs were investigated in detail via TEM and the corresponding results demonstrated in Fig 1. In agreement with results of the average particle size, the TEM observations indicated that nano-ZnOs exhibited almost spherical geometry with diameter about 30-50nm (Fig 1A and 1B). The HR-TEM image showed that these nano-ZnOs were single crystal particles with a lattice constant of around 0.26 nm, as shown in Fig 1C.

### Effects of nano-ZnOs on the body weights

**Fig 2:**
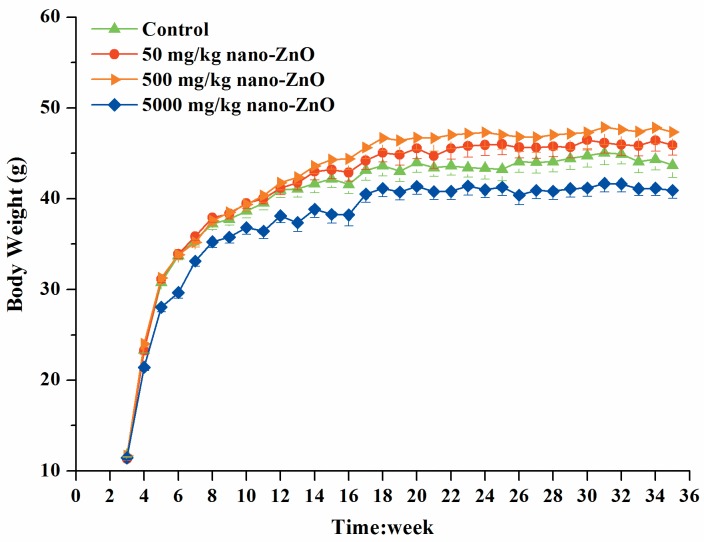
Mean body weights of male mice in control and 50, 500 and 5000 mg/kg groups from week 3 to 35. No. of animals studied per group was 12. For clarity, the significance symbols at different time points are omitted. Mice in 5000 mg/kg nano-ZnO group had significant lower body weights than the control from 4 to 16 weeks; showed lower body weights than 50 and 500 nano-ZnO groups from 4 to 35 weeks. There was no significant difference in body weights of mice among the control, 50 and 500 nano-ZnO groups.

Effects of nano-ZnOs on the body weights were shown in Fig 2. Results indicated that dietary added 5000 mg/kg nano-ZnOs significantly decreased the body weights of male mice at week 4 as compared with those of the control (21.42±0.36 vs 23.34±0.33, *P*<0.01), and this difference lasted for 13 weeks from 4 to 16 week of age. From 17 to 35 week, the body weights were still lower than those of control, while there was no significant difference (*P*>0.05). The addition of 50 and 500 mg/kg nano-ZnOs showed a trend to increase the body weights of mice, while there was no significant difference among the control, 50 and 500 mg/kg nano-ZnO groups (*P*>0.05). However, mice in 50 and 500 nano-ZnO groups showed higher body weights than those in 5000 mg/kg nano-ZnO group from week 4 to 35 (*P*<0.05).

### Effects of nano-ZnOs on the organ weights

Effects of nano-ZnOs on the relative organ weights were present in Table 2. Results indicated that supplementation with doses of nano-ZnOs did not changed the relative weights of liver, spleen, kidney, heart, and testis. However, 5000 mg/kg nano-ZnOs significantly increased the relative weights of the pancreas, brain and lung as compared with the control (*P*<0.05). There was no significant difference in the selected organ weights among the control, 50 and 500 mg/kg nano-ZnO groups (*P*>0.05), except for relative weights of lung which was increased by long term exposure to 500 mg/kg nano-ZnOs (*P* = 0.03).

**Table 2:** Effects of nano-ZnOs on the relative organ weights (10 mg/g).

Item^1^	control	nano-ZnO			SEM^2^	*P*	*P*-Value Trend^3^	
		50 mg/kg	500 mg/kg	5000 mg/kg			L	Q
**liver**	4.17	4.13	4.08	4.11	0.04	0.82	0.45	0.62
**Spleen**	0.23	0.23	0.25	0.27	0.01	0.41	0.21	0.70
**Pancreas**	0.61^b^	0.72^a^^b^	0.73^a^^b^	0.86^a^	0.03	0.01	<0.01	0.88
**Kidney**	1.72	1.75	1.73	1.85	0.03	0.22	0.10	0.35
**Heart**	0.59	0.58	0.59	0.65	0.01	0.09	0.05	0.11
**Testis**	0.79	0.76	0.81	0.82	0.01	0.40	0.21	0.56
**Brain**	1.03^b^	1.05^b^	1.07^b^	1.21^a^	0.02	<0.01	<0.01	0.07
**Lung**	0.67^b^	0.68^a^^b^	0.77^a^	0.78^a^	0.02	0.03	0.01	0.91

^a-b^ Means in a row with different superscripts were significantly different (*P*<0.05).

^1^Data were expressed as mean values.

^2^SEM = standard error of the mean (n = 12).

^3^*P*-Value Trend refers to polynomial trend analysis; L = linear trend; Q = quadratic trend.

### Effects of nano-ZnOs on serum parameters

As shown in Table 3, long term exposure to doses of nano-ZnOs did not affect the serum activities of ALP and GOT (*P*>0.05). However, added 5000 nano-ZnOs significantly increased the zinc concentration and GPT activity in serum as compared to the control (*P*<0.01), while there was no significant difference in serum zinc level and GPT activity among the control, 50 and 500 mg/kg nano-ZnO groups (*P*>0.05).

**Table 3:** Effects of nano-ZnOs on serum parameters.

Item^1^	control	nano-ZnO			SEM^2^	*P*	*P*-Value Trend^3^	
		50 mg/kg	500 mg/kg	5000 mg/kg			L	Q
**Zn(μmol/L)**	25.45^b^	25.15^b^	29.48^b^	38.14^a^	1.37	<0.01	<0.01	0.06
**ALP(U/dL)**	7.69	8.62	7.08	7.51	0.30	0.32	0.43	0.68
**GPT(U/L)**	16.51^b^	17.43^b^	18.84^b^	31.00^a^	1.63	<0.01	<0.01	0.05
**GOT(U/L)**	58.31	54.58	59.18	63.23	1.82	0.43	0.24	0.29

^a-b^Means in a row with different superscripts were significantly different (*P*<0.05).

^1^Data were expressed as mean values.

^2^SEM = standard error of the mean (n = 12).

^3^P-Value Trend refers to polynomial trend analysis; L = linear trend; Q = quadratic trend.

### Effects of nano-ZnOs on mineral contents of selected tissues

Compared with the control, long term exposure to 5000 mg/kg nano-ZnOs significantly (*P*<0.05) increased the Zn contents in the liver, pancreas, kidney, and bones (tibia and fibula), without significant effects on the Zn levels of thigh muscle (rectus femoris muscle and vastus medialis muscle), brain and testis (*P*>0.05), as shown in Table 4. There was no difference in Zn contents of the selected tissues among the control, 50 and 500 mg/kg nano-ZnO groups (*P*>0.05). Long term exposure to nano-ZnOs (50, 500 and 5000 mg/kg) did not affect the contents of Cu, Fe and Mn in selected tissues (*P*>0.05), except for the liver Fe content and pancreas Mn level (*P*<0.05). Addition of nano-ZnOs showed significantly quadratic effects on hepatic Fe contents, which were the highest in mice exposed to 50 mg/kg nano-ZnOs and lowest in mice exposed to 5000 mg/kg nano-ZnOs. As compared to the control, nano-ZnOs (50, 500 and 5000 mg/kg) significantly decreased the Mn contents of the pancreas (*P<*0.05).

**Table 4:** Effects of nano-ZnOs on mineral concentrations of selected tissues.

Item^1^	control	nano-ZnO			SEM^2^	*P*	*P*-Value Trend^3^	
		50 mg/kg	500 mg/kg	5000 mg/kg			L	Q
**Liver**								
Zn(mg/kg)	31.35^b^	31.42^b^	33.26^b^	39.94^a^	0.77	<0.01	<0.01	0.01
Fe(mg/10g)	11.12^a^^b^	14.11^a^	11.87^a^^b^	9.27^b^	0.51	0.01	0.06	<0.01
Cu(mg/kg)	12.21	13.72	13.39	13.75	0.22	0.04	0.03	0.18
Mn(mg/kg)	5.03	4.83	4.69	5.00	0.12	0.75	0.83	0.31
**Pancreas**								
Zn(mg/kg)	29.28^b^	30.67^b^	33.35^b^	211.76^a^	13.31	<0.01	<0.01	<0.01
Fe(mg/kg)	614.29	454.29	489.31	520.03	35.06	0.42	0.44	0.18
Cu(mg/kg)	17.90	15.32	15.97	16.03	0.80	0.71	0.49	0.45
Mn(mg/kg)	11.03^a^	8.50^b^	8.65^b^	9.02^b^	0.30	0.01	<0.01	0.08
**Kidney**								
Zn(mg/kg)	13.63^b^	14.02^b^	14.19^b^	20.42^a^	0.90	0.14	0.01	0.08
Fe(mg/kg)	478.28	403.39	382.19	335.69	22.96	0.17	0.03	0.75
Cu(mg/kg)	12.69	12.34	12.93	12.21	0.25	0.74	0.71	0.72
Mn(mg/kg)	6.40	6.28	6.60	5.47	0.27	0.48	0.31	0.99
**Thigh muscle**								
Zn(mg/kg)	11.98	10.93	10.57	14.39	0.55	0.06	0.15	0.03
Fe(mg/kg)	128.71	130.48	122.97	132.51	4.18	0.88	0.92	0.66
Cu(mg/kg)	4.31	4.58	4.76	5.07	0.12	0.15	0.05	0.91
Mn(mg/kg)	1.26	1.10	1.19	0.93	0.05	0.13	0.06	0.60
**Brain**								
Zn(mg/kg)	7.96	8.35	9.96	9.52	0.64	0.67	0.29	0.76
Fe(mg/kg)	140.22	135.76	162.34	134.76	6.96	0.48	0.87	0.42
Cu(mg/kg)	11.91	11.73	11.42	10.17	0.27	0.08	0.02	0.29
Mn(mg/kg)	1.96	1.91	2.05	1.78	0.10	0.82	0.65	0.61
**Testis**								
Zn(mg/kg)	20.21	23.11	21.20	21.48	0.50	0.23	0.67	0.19
Fe(mg/kg)	229.60^a^^b^	240.99^a^	230.86^a^^b^	202.36^b^	4.74	0.02	0.02	0.03
Cu(mg/kg)	11.83	12.23	11.15	11.98	0.21	0.33	0.74	0.62
Mn(mg/kg)	2.97	3.15	3.02	2.63	0.11	0.36	0.23	0.19
**Bone**								
Zn(mg/kg)	219.41^b^	204.54^b^	261.53^b^	722.77^a^	45.69	<0.01	<0.01	<0.01
Fe(mg/10g)	10.05	10.37	14.26	14.74	1.72	0.70	0.29	0.98
Cu(mg/kg)	12.33	11.81	12.00	14.38	0.37	0.05	0.05	0.04
Mn(mg/kg)	10.95	8.22	10.43	14.09	0.81	0.09	0.10	0.04

^a-b^Means in a row with different superscripts were significantly different (*P*<0.05).

^1^Data were expressed as mean values.

^2^SEM = standard error of the mean(For samples of the liver, pancreas, kidney, thigh muscle, brain and testis, n = 12; for bone samples, n = 6).

^3^P-Value Trend refers to polynomial trend analysis; L = linear trend; Q = quadratic trend.

### Effects of nano-ZnOs on mineral concentrations of feces

Long term exposure to nano-ZnOs did not affect the Fe, Cu and Mn contents in feces (*P*>0.05). However, Added nano-ZnOs significantly increased the fecal Zn contents with significant dose-dependent effects (*P*<0.05), as shown in Table 5.

**Table 5:** Effects of nano-ZnOs on mineral concentrations of feces.

Item^1^	control	nano-ZnO			SEM^2^	*P*	*P*-Value Trend^3^	
		50 mg/kg	500 mg/kg	5000 mg/kg			L	Q
**Zn (mg/10g)**	1.67^c^	4.99^c^	21.21^b^	79.54^a^	9.45	<0.01	<0.01	<0.01
**Fe (mg/10g)**	12.48	8.99	12.27	11.31	76.61	0.06	0.03	0.24
**Cu (mg/kg)**	108.55	92.33	87.76	93.06	5.39	0.61	0.34	0.37
**Mn (mg/kg)**	180.60	176.50	173.54	142.39	8.02	0.35	0.13	0.41

^a-c^Means in a row with different superscripts were significantly different (*P*<0.05).

^1^Data were expressed as mean values.

^2^SEM = standard error of the mean (n = 3).

^3^P-Value Trend refers to polynomial trend analysis; L = linear trend; Q = quadratic trend.

### Effects of nano-ZnOs on the mRNA expression of Zn metabolism-related genes in jejunum

**Fig 3:**
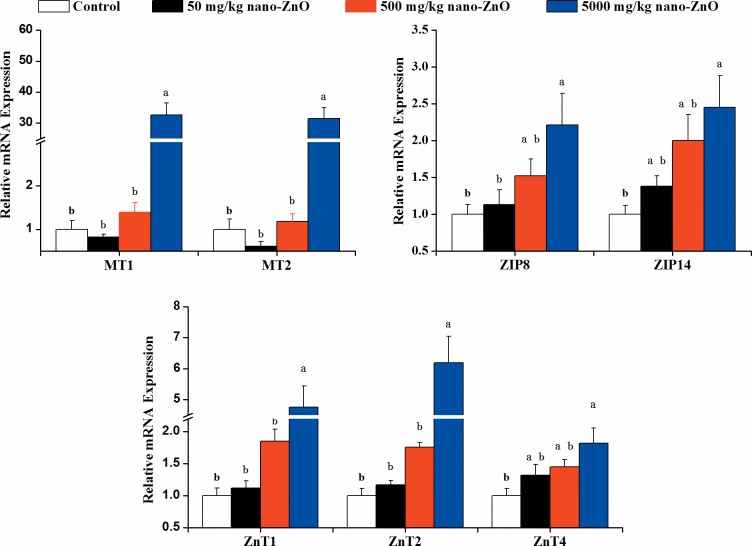
Relative mRNA expression of Zn metabolism-related genes (MT1, MT2, ZIP8, ZIP14, ZnT1, ZnT2, and ZnT4) in jejunum of mice as determined by qPCR. Data expressed relative to the housekeeping gene β-actin, normalized to the NBW group and represent means ± SE (n = 12). ^a-b^ Means for the same gene with different superscripts are significantly different (*P*<0.05).

Compared to the control, mice receiving 5000 mg/kg nano-ZnOs showed significantly (*P*<0.05) higher relative mRNA expression of the selected Zn metabolism-related genes, including MT1 (32.66 folds), MT2 (31.42 folds), ZIP8 (2.21folds), ZIP14 (2.45 folds), ZnT1 (4.76 folds), ZnT2 (6.19 folds) and ZnT4 (1.82 folds). Added 50 and 500 mg/kg nano-ZnOs did not affect the Zn metabolism-related gene expression (*P*>0.05), as shown in Fig 3.

## Discussion

Zinc is an important essential trace element for growth, immune system, metabolism and wound healing in both human and animals. Zinc oxide (ZnO) is generally considered as a cheap inorganic material with low toxicity, which is commonly used as a nutritional or medical additive for the zinc-deficient children or animals [9,22]. Owing to the small size, increased surface area to volume ratio at nanoscale, nano-ZnOs possess multiple properties and have been widely used [4–6]. Although it was reported that nano-ZnOs exhibited high biocompatibility with human cells and high selectivity[27], more concerns have been spurred over the possible adverse effects of exposure to nanoparticles [28,29]. Recently, the acute toxicity of nano-ZnOs had been investigated in vitro and vivo [30–32], while no long term effects of nano-ZnOs had been reported yet.

It has been proved that the size, shape and chemical structure are important factors affect the cytotoxicity caused by nanoparticles [12,33]. The nano-ZnO toxicity was increased with the decreased size or increased concentration [19,21,34]. The cytotoxicity of spherical nano-ZnO was higher than that of the nano-ZnO tetrapods or the flower-like nanoparticle[12,35]. Therefore, the particle size, morphology and chemical structure were first analyzed in the present study. Results of size distribution and TEM images indicated that the average size of nano-ZnOs we used was about 40 nm with a range from 30 to 50 nm. The morphology of these nanoparticles was nearly spherical geometry. The HR-TEM image confirmed that the single crystallinity of these nano-ZnOs exhibited an average lattice spacing of 0.26nm, which was identical to the previous report by Aneesh et al. [7].

Body and organ weights are the common and sensitive indicators for identification of the potentially harmful effects of drugs on animals [36–38]. In this long term study, mice fed with the high dose of nano-ZnOs (5000 mg/kg) had significant lower body weights from week 4 to 16, and tend to be lower from 17 to 35 as compared to the control mice. These results indicated that dietary added high dose of nano-ZnOs could produce toxicological impact, which was in line with previous results reported by Wang et al. [19]. In addition, dietary added 5000 mg/kg nano-ZnOs increased the relative organ weights, including the pancreas, brain, and lung with dose-dependent effects (linear trend). Inconsistent with previous studies, most of the studies reported that nano-ZnOs damaged the organs such as lung, liver and pancreas [18] or decrease their relative weight [22]. The reduced body weight in 5000 mg/kg nano-ZnO group might partially contribute to the increases in the relative organ weights.

The serum biochemical parameters (activities of GOT, GPT, and ALP) were analyzed to evaluate hepatic damage. The activities of GOT and ALP were not changed much, while the activity of GPT, a more sensitive indicator of hepatic injury, was increased after dietary supplementation of 5000 mg/kg nano-ZnOs for 32 weeks. It was reported that the liver is a main target organ damaged by nano-ZnOs. Hong et al. [22] revealed that 400 mg/kg/day nano-ZnOs significantly decreased the absolute liver weight of dams. Wang et al. [19] reported that 14-d exposure to high dose of nano-ZnOs (1–5 g/kg body weight) led to hepatic damages aggravated with the dose increase by histopathologic examination. The possible mechanism for hepatic injury might be involved in the zinc accumulation of nano-ZnOs, which further induced the oxidative stress, DNA damage and apoptosis in liver [30].

The biodistribution of zinc was also investigated in this study. Long term exposure to 5000 mg/kg nano-ZnOs did not affect the zinc level in muscle, brain, and testis, but increased the zinc contents in liver, pancreas, kidney and bones. Wang et al. [19] also revealed that zinc was mainly retained in the bone, kidney and pancreas after 20-nm and 120-nm ZnO administration. The biodistribution of nanoparticles in the tissues indicated that particles could be transported to other tissues and organs after uptake by gastrointestinal tract [24]. However, this study just determined the zinc content for the biodistribution, and the levels of nano-ZnOs in different tissues or organs should be analyzed in future. It was reported that zinc affected the absorption and biodistribution of other minerals, including Cu, Fe and Mn [39,40]. However, there was no effects of dietary nano-ZnOs (50, 500 and 5000 mg/kg) on the Cu, Fe, and Mn contents in selected tissues, except for the liver Fe content and pancreas Mn level.

Fecal, serum zinc contents and expression of zinc metabolism-related genes were used to determine the long term effects of nano-ZnOs on the zinc metabolism. Dietary supplemented 5000 mg/kg nano-ZnOs increased the serum zinc level, indicating that dietary supplementation with high dose of nano-ZnOs increased zinc absorption, while decreased the zinc bioavailability since the fecal zinc contents significantly increased as the dietary nano-ZnO level increase. Gastrointestinal system is a central system for zinc homeostasis and the small intestine charges for the dietary zinc absorption and intestinal zinc excretions [41–43]. Hoadley et al. [44] revealed that metallothionein (MT) acts as a zinc pool, from which zinc is highly available to be transported back to the lumen. The absence of MT resulted in a detectable increase in zinc accumulation in the small intestine [45]. Four isoforms of MT have been described, while MT1 and MT2 are highly expressed in the small intestine [46]. In this study, long term exposure to 5000 mg/kg nano-ZnOs significantly enhanced the MT1 and MT2 mRNA expression (32.66 and 31.42 folds, respectively), indicating that dietary added 5000 mg/kg nano-ZnOs might transport zinc back to the intestinal lumen, which could partially explain the increased fecal zinc contents.

Although most of the studies have found that oral treatment with nano-ZnOs could enhance the zinc absorption and tissue distribution[19,47], the mechanism is still unclear. It has been thought that nanoparticles could permeate through the gastrointestinal barrier and the neutral surface charge increased this diffusion rate[13,48]. However, WG et al. [49] evidenced that the insoluble nanoparticles could not be absorbed in the gut, who also speculated that in contrast to the insoluble iridium, the zinc oxide nanoparticles was much more soluble and could be absorbed as zinc ions. In our present study, the mRNA expression of ZIP and ZnT families were determined, which are the two main conserved families regulating the process of intestinal zinc uptake [42,50]. Our present results showed that dietary added 50 and 500 mg/kg nano-ZnOs did not change the mRNA expression of ZIP family (ZIP 8 and 14) and ZnT family (ZnT1, 2 and 4), while 5000 mg/kg nano-ZnOs enhanced the mRNA expression of these genes. ZIP8 and ZIP14 are abundantly expressed in small intestine and localized to the apical membrane of polarized cells, and mediate the intestinal zinc into the enterocytes [51,52]. Knockout of ZIP14 significantly reduced the cellular zinc contents in chondrocytes [52]. ZnT1 and ZnT4 are abundant in the proximal small intestine and directly mediates zinc efflux across the basolateral membrane [53,54]. ZnT2 is also expressed in small intestine, which localized to vesicles on the apical side of the enterocytes [55]. Our results indicated that added high dose of nano-ZnO enhanced the intestinal zinc absorption, which might be, at least partially, attributed to the absorption of zinc ions via regulating both ZIP and ZnT families [42,49].

## Conclusions

In summary, the high crystalline quality of nano-ZnOs (about 40nm) were used here to investigate the long term effects on the development, zinc metabolism and mineral biodistribution (Cu, Zn, Fe and Mn) in male mice. These results indicated that long term exposure to 50 and 500 mg/kg nano-ZnOs showed minimal toxicity to male mice from week 3 to 35, while 5000 mg/kg nano-ZnOs decreased the body weight, increased the relative weights of the pancreas, brain and lung. Dietary added 5000 mg/kg nano-ZnOs damaged hepatic function, altered zinc metabolism in small intestine and led to a significant accumulation of zinc in the liver, pancreas, kidney, and bones (tibia and fibula), without significant effects on Cu, Fe, and Mn levels, except for the liver Fe content and pancreas Mn level. This study just analyzed the zinc content in the biodistribution assays, and levels of nano-ZnOs in different tissues or organs should be investigated in future. Our comprehensive observations might be helpful in reducing the excessive worry about oral exposure to low level of dietary nano-ZnOs. These results could also be beneficial in the foreseen researches of nano-ZnOs and their applications in agriculture and biomedicine. It is better to study the long term effects of nanoparticles with some more suitable animal models, such as the model of pigs.
